# Investigating iRHOM2-Associated Transcriptional Changes in Tylosis With Esophageal Cancer

**DOI:** 10.1016/j.gastha.2023.12.007

**Published:** 2023-12-26

**Authors:** Stephen Murtough, Deepak Babu, Catherine M. Webb, Hélène Louis dit Picard, Lisa A. McGinty, Jennifer Chao-Chu, Ryan Pink, Andrew R. Silver, Howard L. Smart, John K. Field, Philip Woodland, Janet M. Risk, Diana C. Blaydon, Daniel J. Pennington, David P. Kelsell

**Affiliations:** 1 Faculty of Medicine and Dentistry, Blizard Institute, Queen Mary University of London, London, UK; 2 Department of Biological and Medical Sciences, Faculty of Health and Life Sciences, Oxford Brookes University, Oxford, UK; 3 Gastroenterology and Liver Services, Liverpool University Hospitals NHS Foundation Trust, Liverpool, UK; 4 Department of Molecular and Clinical Cancer Medicine, Institute of Systems, Molecular and Integrative Biology, University of Liverpool, Liverpool, UK; 5 Endoscopy Unit, Barts Health NHS Trust, The Royal London Hospital, London, UK

**Keywords:** iRhom2, ESCC, Tylosis With Esophageal Cancer, RNA-Seq, Early Cancer Detection

## Abstract

**Background and Aims:**

Survival rates for esophageal squamous cell carcinoma (ESCC) are extremely low due to the late diagnosis of most cases. An understanding of the early molecular processes that lead to ESCC may facilitate opportunities for early diagnosis; however, these remain poorly defined. Tylosis with esophageal cancer (TOC) is a rare syndrome associated with a high lifetime risk of ESCC and germline mutations in *RHBDF2*, encoding iRhom2. Using TOC as a model of ESCC predisposition, this study aimed to identify early-stage transcriptional changes in ESCC development.

**Methods:**

Esophageal biopsies were obtained from control and TOC individuals, the latter undergoing surveillance endoscopy, and adjacent diagnostic biopsies were graded as having no dysplasia or malignancy. Bulk RNA-Seq was performed, and findings were compared with sporadic ESCC vs normal RNA-Seq datasets.

**Results:**

Multiple transcriptional changes were identified in TOC samples, relative to controls, and many were detected in ESCC. Accordingly, pathway analyses predicted an enrichment of cancer-associated processes linked to cellular proliferation and metastasis, and several transcription factors were predicted to be associated with TOC and ESCC, including negative enrichment of GRHL2. Subsequently, a filtering strategy revealed 22 genes that were significantly dysregulated in both TOC and ESCC. Moreover, Keratin 17, which was upregulated in TOC and ESCC, was also found to be overexpressed at the protein level in ‘normal’ TOC esophagus tissue.

**Conclusion:**

Transcriptional changes occur in TOC esophagus prior to the onset of dysplasia, many of which are associated with ESCC. These findings support the utility of TOC to help reveal the early molecular processes that lead to sporadic ESCC.

## Introduction

Most esophageal squamous cell carcinomas (ESCCs) are diagnosed late, when the disease is advanced and has spread from its primary site.^1^ This considerably affects the prognosis and chance of survival, and stage at diagnosis remains the most important prognostic factor.^2^ Yet, early-stage ESCCs rarely present with symptoms, and no screening program for at-risk individuals, such as tobacco smokers and alcohol drinkers, exists. Accordingly, an opportunity to improve early diagnosis of ESCC may be to use biomarkers of early-stage disease, although the molecular changes underpinning early-stage disease remain poorly characterized, in part due to a lack of useful models to identify these changes.

Tylosis with esophageal cancer (TOC) is a rare, autosomal dominant syndrome associated with late-onset focal nonepidermolytic palmoplantar keratoderma, germline mutations in *RHBDF2* (encoding iRhom2), and a high lifetime risk of ESCC, estimated to be 90% by 70 years.^3^^,^^4^ Additionally, *RHBDF2* mutations in TOC represent the only known genetic susceptibility that leads to ESCC with high penetrance, and TOC may therefore be a useful model to identify early carcinogenic processes in the esophagus. To date, studies into the biology of TOC have largely focused on the skin,^5^, ^6^, ^7^ and no molecular description of TOC esophagus exists.

iRhom2 is a highly conserved, catalytically inactive Rhomboid protease associated with an array of biological functions.^8^ These include facilitating the maturation and trafficking of ADAM17, which is a sheddase of multiple ligands, including those involved in the epidermal growth factor receptor (EGFR) pathway, and inflammatory cytokines, such as tumor necrosis factor-alpha (TNF-α).^9^^,^^10^ In hyperproliferative epidermal keratinocytes, such as those isolated from TOC individuals, the shedding of ADAM17 ligands and associated EGFR signaling is increased.^5^^,^^11^ Moreover, iRhom2 associates with the stress keratin, Keratin 16,^6^ and is a transcriptional target for p63,^7^ in the keratinocyte stress response. However, the biology of iRhom2 in esophageal carcinogenesis remains largely unexplored and is of importance given the role of iRhom2 as a familial esophageal squamous cancer gene.

Genome-wide transcriptomics studies have shown great utility in revealing important aspects of the disease, and the ESCC transcriptome has been well explored with microarray,^12^^,^^13^ bulk RNA sequencing (RNA-Seq),^14^, ^15^, ^16^ and single-cell RNA-Seq^17^^,^^18^ studies. How the transcriptome is altered in predysplastic and premalignant esophageal epithelium remains unexplored, however, and may provide insight into the biology underpinning these early stages of disease. Although to date, there have been difficulties in determining which early-stage biopsies are high risk for ESCC, impeding these types of studies.

Here, bulk RNA-Seq of early-stage TOC and normal esophageal biopsies reveals the transcriptional landscape of TOC esophagus and bioinformatics and comparison strategies uncover similarities between TOC and sporadic ESCC. Moreover, these data provide insight into the early-stage processes underlying sporadic ESCC and represent a springboard for future investigations using TOC as a model of early-stage esophageal disease.

## Materials and Methods

### Patient Samples

**Figure 1:**
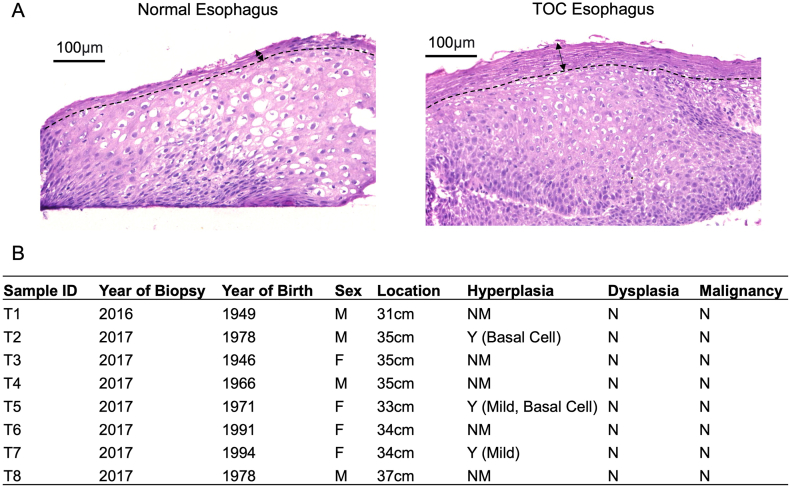
Histological summary of TOC esophageal biopsies (A) Representative H&E images of normal and TOC esophagus tissue. Dotted line is drawn to indicate beginning of superficial terminally differentiated cell layer, and arrows are shown to indicate width of this tissue region. Scale = 100 μm. (B) Pathology reports for biopsies taken at adjacent regions in the esophagus to samples processed for bulk RNA-Seq. Pathology notes were summarized according to relevant histological criteria. N, no; NM, not mentioned; Y, yes.

Esophageal biopsies were collected from 8 UK TOC family members with confirmed *RHBDF2* mutation status (c.557 T>C, p.Ile186Thr), during surveillance endoscopy (REC 02/8/001). Pathologist reports for adjacent diagnostic biopsies taken at the same level of the esophagus were provided, and these are summarized in the Results section, Figure 1B. Six normal esophageal biopsies were collected from 2 volunteers who did not have a history of esophageal disease, nor any signs of esophageal erosion and/or esophagitis during endoscopy, and details of these are described in Table A1. (REC 15/LO/2127). Given that the normal esophageal biopsies were limited to 2 patients, we validated the representative clustering with an additional 8 normal patient biopsies (data not shown^19^).

### Bulk RNA-Sequencing

#### Data generation

Biopsies for bulk RNA-Seq were collected in RNAlater RNA Stabilizing Solution (Invitrogen), and RNA was extracted and processed by Queen Mary University of London’s Genome Centre. Polyadenylated mRNAs were captured using NEBNext Poly(A) mRNA Magnetic Isolation Module (New England BioLabs), and the NEBNext Ultra II Directional RNA Library Prep Kit for Illumina (New England BioLabs) was used to create cDNA libraries, which were sequenced using the NextSeq 500 System (Illumina).

#### Data processing

Paired-end FASTQ files were uploaded to the RaNA-seq server,^20^ and Salmon^21^ was used to align files to the GRCh38 human genome to produce gene-level expression estimates. Additionally, publicly available RNA-Seq datasets were imported into RaNA-seq via their Sequence Read Archive accession number, and gene-level expression estimates were generated using Salmon as described above.

#### Differential gene expression analysis

DESeq2^22^ was used to identify differentially expressed genes from raw gene-level counts. Differentially expressed genes were identified by filtering by log_2_ fold change, adjusted *P*-value, log_2_ fold change standard error and mean normalized count (as Transcripts per Million [TPM]).

#### Clustering and dimensionality reduction analysis

Hierarchical clustering was performed on differentially expressed genes to limit noise from lowly expressed and/or nonvariable genes, and TPM counts for these were scaled and centered, and then clustered with hclust (R Stats Package, v.4.3.1). Clustering dendrograms were visualized as part of a heatmap with the pheatmap package (v.1.0.12). To perform principal components analysis (PCA), lowly expressed genes were removed, and the prcomp function (R Stats Package, v.4.3.1) was used to scale and center TPM counts and to generate PCA scores. Biplots of principal component 1 and principal component 2 were visualized using ggplot2 (v.3.4.3).

#### Pathway enrichment analysis

Lists of differentially expressed genes were processed by the Enrichr online server,^23^, ^24^, ^25^ to identify enriched GO Biological Process, Cellular Component, and Molecular Function terms; BioPlanet terms; and Human Phenotype Ontology terms. Terms with a false discovery rate (FDR) <0.05 were selected. Additionally, raw gene-level counts were normalized by variance stabilizing transformation by DESeq2, and lowly expressed genes were removed prior to input into the gene set enrichment analysis (GSEA) software application (v.4.3.2).^26^^,^^27^ GSEA was performed using the Hallmark Gene Sets database to determine enriched biological pathways. Terms with a *P*-value <.05 and FDR <0.25 were selected.

#### Transcription factor prediction analysis

Transcription factor regulons were obtained from the curated DoRothEA R package (v.1.12.0) for human (dorothea_hs).^28^ The msviper function (VIPER R package, v.1.34.0) was used in conjunction with these regulons to calculate positively and negatively enriched transcription factors. Transcription factors with a confidence score of A–D were used as input, and those with a normalized enrichment score >1.5 or <−1.5, and *P*-value <.05 were selected.

### Statistics

Statistical tests were applied to data throughout the analysis pipeline. The differential gene expression package, DESeq2, computes adjusted *P*-values, which were used to select significant genes. TPM values were log_2_+1 transformed to normalize data and avoid negative values. Two-sample *t*-tests with FDR correction were applied to data using the rstatix package (v.0.7.2), and adjusted *P*-values were displayed as significance stars on plots.

### Quantitative PCR

RNA from samples used for bulk RNA-Seq was used for confirmatory quantitative polymerase chain reaction (qPCR) reactions. RNA was converted to cDNA using the high-capacity cDNA reverse transcription kit (ThermoFisher Scientific, catalog number: 4368814) with supplementation of an RNase inhibitor (ThermoFisher Scientific, catalog number: N8080119). qPCR reactions were performed in triplicate in 96-well PCR plates (Starlab, catalog number: E1403-7700). Each well-contained 10μl of TaqMan Gene Expression Master Mix (ThermoFisher Scientific, catalog number: 4369510), 1μl of TaqMan Assay (ThermoFisher Scientific, catalog number: 4331182) for a specific gene target (Table A2), and 9μl of sample cDNA at 10ng/μl. Glyceraldehyde 3-phosphate dehydrogenase was used as a housekeeping gene, and no-template-control wells, containing 9μl of nuclease-free water in place of cDNA, were run for each TaqMan Assay mix. Plates were centrifuged and run using a StepOnePlus Real-Time PCR System (ThermoFisher, catalog number: 4376598), according to the manufacturer’s instructions. Gene expression was calculated relative to mean C_t_ values for glyceraldehyde 3-phosphate dehydrogenase and to average expression values across control (normal esophagus) samples using the 2^−ΔΔCt^ method.

### Immunofluorescence Staining

Tissue sections were obtained from OCT-embedded frozen tissue, thawed at room temperature for ∼20 minutes, and washed 3 times with phosphate-buffered saline (PBS). Sections were fixed either with 4% paraformaldehyde (PFA) or 50:50 Methanol Acetone, depending on primary antibody, for 15 minutes at room temperature, washed 3 times with PBS (and washed for an extra 10 minutes with PBS and 0.1% Triton, in sections fixed with 4% PFA), incubated with 5% goat serum in PBS for one hour at room temperature, and incubated overnight at 4 °C with primary antibody (GRHL2, HPA004820, 1 in 100 dilution, tissue fixed with 4% PFA; Keratin 17, ab51056, 1 in 300 dilution, tissue fixed with 50:50 Methanol Acetone) diluted in 5% goat serum in PBS. The following day, sections were washed 3 times with PBS, incubated with goat anti-rabbit IgG Alexa Fluor 488 secondary antibody (ThermoFisher Scientific, catalog number: A-11008) for one hour at room temperature, washed 3 times with PBS, incubated with 4′,6-diamidino-2-phenylindole (DAPI) (diluted 1 in 10,000 in PBS) for 10 minutes at room temperature, washed 3 times with PBS, and mounted using ImmuMount (Epredia). Sections were imaged on an upright Leica DM4000 Epi-Fluorescence Microscope, and images were quantified using Fiji software.

### Hematoxylin and Eosin Staining

Formalin-fixed paraffin embedded tissue blocks were sectioned and stained for hematoxylin and eosin by Queen Mary University of London’s Pathology Services.

### Datasets Accessed for This Study

Publicly available ESCC vs normal RNA-Seq datasets were accessed via Sequence Read Archive using their accession numbers: SRP008496, SRP064894, and SRP133303.

## Results

### TOC Esophageal Tissue Displays Abnormal Histological Features Prior to Dysplasia

‘Normal’ (predysplasia) TOC esophageal tissue was found to display histological differences to *bona fide* normal esophageal tissue, including a thickened superficial layer that appeared to be keratinized, suggesting an altered keratinocyte differentiation program and barrier function (Figure 1A). Moreover, this hinted that the hyperactive iRhom2 mutant may have driven these histological changes prior to the development of dysplasia in TOC esophageal tissue.^29^^,^^30^

To explore the molecular pathways underlying these histological changes, bulk RNA-Seq was performed. Eight esophageal biopsies were obtained from 4 male and 4 female TOC individuals with confirmed *RHBDF2* mutation status (c.557 T>C, p.Ile186Thr). Pathology reports (summarized in Figure 1B) for adjacent diagnostic biopsies taken at the same level of the esophagus revealed that none were determined to have dysplasia or malignancy, and just 3 adjacent samples were graded as having hyperplasia. While the biopsies assessed in this project were not the exact biopsies referred to in the pathology reports, our assumption was that their histological attributes were likely to be similar given their proximity to adjacent diagnostic biopsies and given that these patients were not recommended for further treatment or close follow-up surveillance. Moreover, given these pathology findings, it was assumed that these biopsies were likely to be predysplastic and therefore be useful to reveal early molecular changes underpinning the development of ESCC.

### The Transcriptome in Early-Stage TOC Esophagus Diverges From a Normal Esophagus State

**Figure 2:**
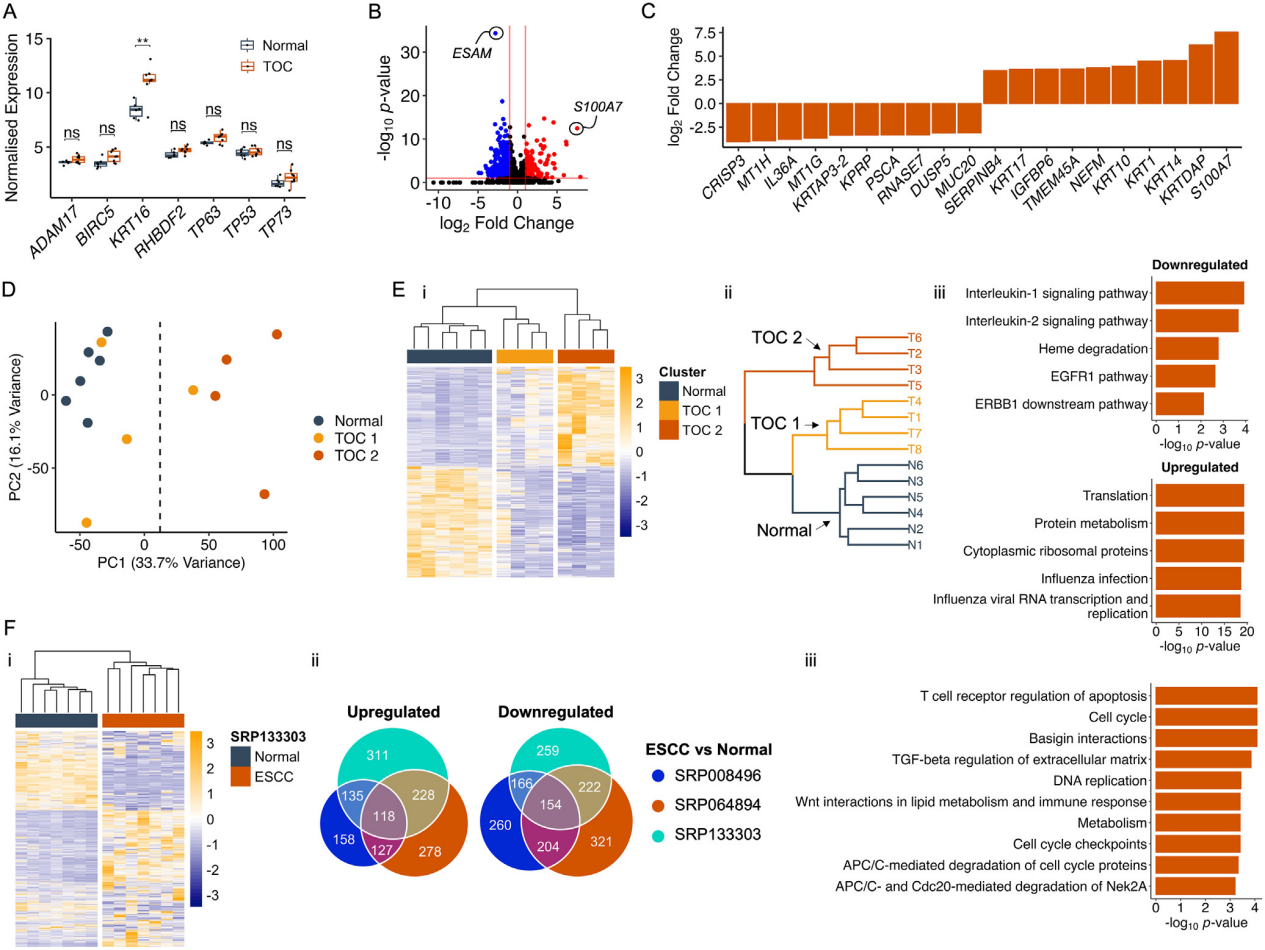
Early-stage TOC esophageal biopsies display a gene expression profile that is distinct from a normal esophagus state and is transcriptionally similar to ESCC. (A) Boxplots showing expression counts (as log2 [TPM+1]) in TOC and normal esophageal samples for 7 genes associated with TOC and iRhom2. Adjusted *P*-values are shown, and statistical significance was calculated using a two-sample t-test with the rstatix package (v.0.7.2). ns = not significant, ∗∗*P* < .01. A plot was produced using ggplot2 (v.3.4.3). (B) Volcano plot showing differentially expressed genes between TOC and normal esophageal samples. Before plotting, differential gene expression matrices were filtered for genes with mean TPM count >5. Vertical red lines indicate log2 fold change of −1 and 1, and the horizontal red line indicates an adjusted *P*-value of .1. Black points denote nonsignificant genes, blue points denote downregulated genes, and red points denote upregulated genes. (C) Bar plot showing the top 10 upregulated and top 10 downregulated genes (ranked by log2 fold change) in TOC esophagus samples, relative to normal esophagus controls. (D) PCA plot of PC1 and PC2, calculated using 9829 genes with mean TPM count >5. A vertical dotted line is shown, indicating a split of samples along PC1. Principal components were calculated using base R’s stats package (v.4.3.1). (E) (i) Heatmap of TPM values for 1241 differentially expressed genes (identified in TOC vs normal samples by: log2 fold change ≥0.58 or ≤−0.58 (fold change of 1.5), adjusted *P*-value <.1, and mean TPM value >10) in TOC vs normal samples; (ii) clustering dendrogram, color-coded according to normal, TOC 1, and TOC 2 transcriptional clusters; and (iii) top 5 enriched BioPlanet pathways (ranked by adjusted *P*-value) associated with downregulated and upregulated genes used to identify the TOC 1 and TOC 2 transcriptional clusters. Heatmap produced using pheatmap package in R (v.1.0.12); dendrogram produced using hclust (R Stats Package, v.4.3.1) and visualized using dendextend (v.1.17.1) and ggplot2 (v.3.4.3); and gene ontology bar plot produced using ggplot2 (v.3.4.3). (F) (i) Heatmap of TPM values for genes identified in part Ei, in ESCC and normal samples (SRP133303). (ii) Euler diagrams showing how many of the 1241 genes are significantly upregulated or downregulated in ESCC samples and how many are shared between 3 publicly available ESCC vs normal datasets; SRP008496, SRP064894, and SRP133303. (iii) Overrepresented BioPlanet pathways associated with the 118 common upregulated genes in ESCC samples. Heatmap produced using pheatmap package in R (v.1.0.12); Euler diagrams produced using eulerr package in R (v.7.0.0); gene ontology bar plot produced using ggplot2 (v.3.4.3). PC1, principal component 1; PC2, principal component 2.

Initial investigations found that several iRhom2-associated genes displayed a trend of being, or were significantly, upregulated in TOC esophagus samples, including *BIRC5* (encoding the anti-apoptotic protein, SURVIVIN), *KRT16*, *RHBDF2*, and *TP63* (Figure 2A). Conversely, *ADAM17* gene expression was comparable to normal esophagus samples (Figure 2A); of note, prior work has shown that ADAM17 protein activity is increased in TOC skin rather than transcript abundance.^5^ Additionally, *TP53* and *TP73* were assessed given their homology to *TP63* and links to cancer^31^; however, their gene expression was unaltered (Figure 2A). Differential gene expression analysis revealed >500 significantly altered gene transcripts between TOC and normal esophagus samples, including upregulation of S100A7, which has been reported to be elevated in ESCC,^32^ and downregulation of *ESAM* (Figure 2B). A closer look at the protein-coding genes with the largest fold change revealed an elevation of a variety of keratin genes, including *KRT14*, *KRT10*, and the differentiation-associated protein, *KRTDAP*, and was suggestive of an abnormal keratinocyte differentiation program (Figure 2C).

To assess for trends and similarities within the data, PCA was performed, and according to principal component 1 (which accounted for 33.3% of variability within the dataset), 3 TOC samples grouped more closely to normal samples, while the other 5 TOC samples grouped further away, suggesting transcriptional variation between the TOC samples (Figure 2D). To investigate further, unsupervised hierarchical clustering was performed on 1241 differentially expressed genes, and this revealed a similar clustering pattern, where 4 TOC samples clustered closely to normal samples and were termed TOC 1, and 4 TOC samples clustered further away and displayed an opposite pattern of gene expression and were termed TOC 2 (Figure 2Ei). Moreover, these groupings did not correlate with age or pathology criteria, such as hyperplasia (Figure 2Eii). Given that the expression of these 1241 genes were able to separate TOC and normal samples, these were investigated by pathway analysis, and upregulated genes (in TOC samples) were associated with protein translation pathways, and downregulated genes (in TOC samples) were associated with several signaling pathways including interleukin-1, interleukin-2, and EGFR1 (Figure 2Eiii). Moreover, the latter was not expected given that we have shown elevated EGFR signaling, including ADAM17 shedding of EGF ligands, in TOC skin,^5^^,^^11^ suggesting that tissue context is important to understanding TOC esophageal biology. Given that the transcriptome in the TOC 2 sample group deviated from a normal esophagus state, which may suggest progression of disease, the expression of these 1241 genes were then assessed in 3, independent publicly available sporadic ESCC vs normal RNA-Seq datasets: SRP008496, SRP064894, and SRP133303. Visualization of these genes via heatmaps revealed that normal and ESCC samples clustered separately, and ESCC samples displayed a visually similar gene expression pattern to TOC 2 samples (Figure 2Fi and Figure A1), suggesting similarity between the TOC 2 sample group and ESCC, albeit without major changes to their histology. Additionally, many of these genes were differentially expressed across all 3 ESCC vs normal datasets, including 118 upregulated and 154 downregulated genes (Figure 2Fii). To investigate these shared genes as transcriptional changes of interest, pathway analysis revealed that the 118 upregulated genes were predominantly associated with cellular proliferation and cell cycle pathways, suggesting that cell turnover in TOC esophageal tissue is elevated (Figure 2Fiii).

### Transcriptional Analyses Reveal Skin and Cancer-Associated Processes in Early-Stage TOC Esophagus

**Figure 3:**
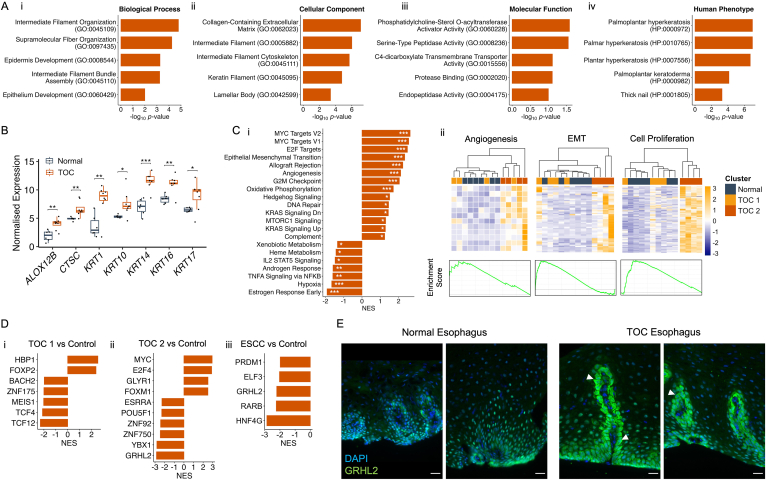
Pathway and transcription factor prediction analyses reveal early molecular changes in TOC that reflect PPK and ESCC. (A) Top 5 (ranked by adjusted *P*-value) overrepresented Gene Ontology pathways, split by (i) Biological Process, (ii) Molecular Function, (iii) Cellular Component, and (iv) Human Phenotype Ontology terms, associated with 88 upregulated genes in TOC esophageal samples relative to control samples, identified by log2 fold change >1.5, adjusted *P*-value <.1, log2 fold change standard error <1, and mean TPM value across samples >5. Bar plots produced using ggplot2 (v.3.4.3). (B) Boxplots showing expression counts (as log2 [TPM+1]) in TOC and normal esophageal samples for 7 genes linked to the Palmoplantar Keratoderma Human Phenotype Ontology [HP:0000982] that were identified as being upregulated in TOC esophageal samples. Adjusted *P*-values are shown. Statistical significance was calculated using a two-sample t-test with the rstatix package (v.0.7.2). ∗*P* < .05, ∗∗*P* < .01, ∗∗∗*P* < .001. Plot produced using ggplot2 (v.3.4.3). (C) (i) Bar plots showing enriched GSEA hallmark gene sets (*P* < .05 and FDR <0.25) in TOC esophageal samples, relative to controls. (ii) Heatmaps showing expression of core-enriched genes associated with angiogenesis, epithelial to mesenchymal transition (EMT), and cell proliferation (collated across MYC targets V1 and V2, E2F Targets, and G2M Checkpoint pathways) and color-coded according to normal, TOC 1, and TOC 2 transcriptional clusters. Beneath each heatmap, GSEA enrichment plots are shown, and the associated plot for MYC targets V2 is shown as representation for cell proliferation. Enrichment was performed using GSEA software (v.4.3.2). Significance scores are denoted as ∗*P* < .05, ∗∗*P* < .01, ∗∗∗*P* < .001. Plots were produced using pheatmap (v.1.0.12) and ggplot2 (v.3.4.3). (D) Bar plots showing NES for enriched transcription factors in (i) TOC1, (ii) TOC 2, and (iii) ESCC esophageal samples. Transcription factors enriched in ESCC were assessed and satisfied across SRP008496, SRP064894, and SRP133303 datasets. Predictions were calculated using the VIPER algorithm (v.1.34.0) and the human DoRothEA-curated regulon, dorothea_hs (v.1.12.0), for transcription factors with confidence scores, (A–D). (E) Representative immunofluorescence images of GRHL2 in normal and TOC esophagus tissue. GRHL2 is shown in green; DAPI nuclei stain is shown in blue; white arrows indicate examples of cytoplasmic/perinuclear staining; magnification = ×40; scale bar = 25μm. NES, normalized enrichment score.

Gene ontology (GO) analyses of significantly upregulated genes revealed an enrichment of skin-associated pathways in TOC esophageal biopsies, including epidermis development (Figure 3Ai), lamellar body formation (Figure 3Aii), and serine-type peptidase activity (Figure 3Aiii). Human phenotypes associated with these upregulated genes were pathways linked to palmoplantar keratoderma (PPK) and hyperkeratosis, which are surprising data given that these genes were identified in esophageal tissue, although, of course, PPK is part of the TOC syndrome. We then investigated which genes were driving these enrichment results, and it was found that these included several keratin genes as well as others commonly associated with skin disease (Figure 3B). Separate pathway analyses were also carried out including GSEA, which considers actual gene expression values. These revealed a positive enrichment (overrepresentation) of several pathways commonly associated with cancer, including those linked to cellular proliferation such as MYC (which is a proto-oncogene and transcription factor that is amplified in several cancers) targets and G2M checkpoint, angiogenesis, and epithelial mesenchymal transition, the latter being associated with invasion and metastasis (Figure 3Ci). Moreover, negatively enriched (underrepresented) GSEA pathways in TOC samples included TNF-α signaling, which again contrasts with previously published data that found this pathway to be elevated in TOC skin via the iRhom2-ADAM17 pathway^5^; as well as endocrine pathways, androgen response, and estrogen response early (Figure 3Ci). We investigated the cancer-associated pathways further by visualizing the expression of their core leading-edge genes (identified by GSEA software), and it was found that these genes were almost exclusively transcriptionally elevated in the TOC 2 group of samples, while TOC 1 samples generally clustered alongside normal esophageal samples and displayed a similar expression pattern (Figure 3Cii).

To explore which transcription factors may be facilitating these transcriptional changes, we made use of the DoRothEA suite of curated regulons, which are a catalog of transcription factors and their gene targets.^28^ To improve robustness and confidence in the output, we discarded transcription factors with a confidence score of *E*, which is DoRothEA’s lowest confidence score and includes transcription factors mapped to gene targets using just one information source. These analyses predicted that different transcription factors regulated the transcriptional changes observed in TOC 1 and TOC 2 samples (Figure 3Di and ii). Given the similarities previously identified between TOC 2 and ESCC samples, further analysis was performed on 3 ESCC vs normal RNA-Seq datasets (SRP008496, SRP064894, and SRP133303). Common transcription factors across all datasets were identified, which revealed some similarities between TOC 2 and ESCC, including negative enrichment of genes linked to the transcription factor GRHL2 (Figure 3Dii and iii). Exploring further, GRHL2 was found to have reasonable gene expression and was selected for further investigation as a transcription factor of interest. Immunofluorescence staining of GRHL2 revealed a nuclear staining pattern in normal esophagus tissue, while in TOC esophagus tissue, GRHL2 staining displayed a perinuclear pattern in papillae structures (Figure 3E and Figure A2). Therefore, it appears that GRHL2 can accumulate outside of the nucleus in these regions of TOC esophageal tissue, which supports the bioinformatics predictions of a negative enrichment of GRHL2 target genes.

### Significant Transcriptional Changes Are Shared Between Early-Stage TOC Esophagus and Sporadic ESCC

**Figure 4:**
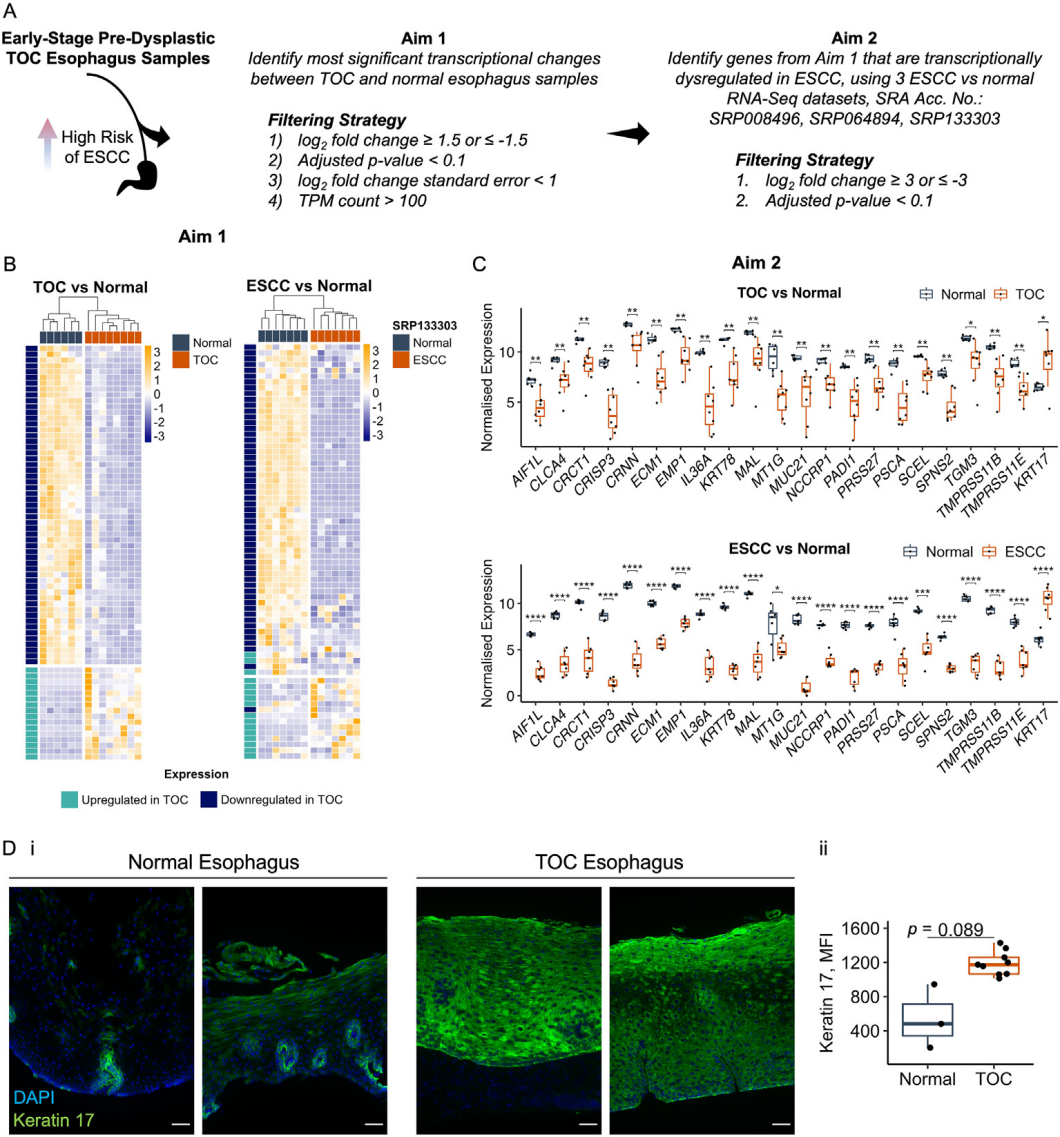
A comparison of early-stage TOC and ESCC RNA-Seq datasets reveals 22 genes that are commonly dysregulated. (A) Schematic describing the filtering strategy that was designed to identify significant genes and to compare TOC and ESCC RNA-Seq datasets. (B) Heatmaps showing TPM counts for 71 genes that were identified according to Aim 1, described in part A, in TOC and normal, and ESCC and normal (SRP133303) esophageal samples. Heatmaps are produced using pheatmap (v.1.0.12). Normal samples are annotated with dark green-blue; TOC and ESCC samples are annotated with orange; genes downregulated in TOC are annotated with dark blue; and genes upregulated in TOC are annotated with green. (C) Boxplots showing expression counts (as log2 [TPM+1]) for 22 genes identified according to Aim 2, outlined in part A, in TOC and normal, and ESCC and normal (SRP133303) esophageal samples. Adjusted *P*-values are shown, and statistical significance was calculated using a two-sample *t*-test with the rstatix package (v.0.7.2). ∗*P* < .05, ∗∗*P* < .01, ∗∗∗*P* < .001, ∗∗∗∗*P* < .0001. Plots were produced using ggplot2 (v.3.4.3). (D) (i) Representative immunofluorescence images of Keratin 17 in TOC and normal esophagus tissue. Keratin 17 is shown in green; DAPI nuclei stain is shown in blue; magnification = x20; scale bar = 50μm. (ii) Boxplot showing quantification of mean fluorescence intensity of Keratin 17 staining in 3 control and 9 TOC esophagus sections, calculated using FIJI software (v.2.1.0). Statistical significance was calculated using a two-sample *t*-test with the rstatix package in R (v.0.7.2).

Considering that several of our analyses identified similarities between predysplastic TOC and sporadic ESCC biopsies, we sought to identify the most significant shared transcriptional changes. To do so, a two-step filtering strategy was designed: first, to identify the most significant changes between TOC and normal esophagus samples, differential gene expression lists were filtered by log_2_ fold change ≥1.5 or ≤−1.5, adjusted *P*-value <.1, log_2_ fold change standard error <1, TPM count >100 (averaged as mean across TOC samples when identifying upregulated genes, and across normal samples when identifying downregulated genes); and second, we aimed to filter genes that were identified in the first filtering run for differentially expressed genes that were satisfied across 3 sporadic ESCC vs normal RNA-Seq datasets (SRP008496, SRP064894, SRP133303), with the criteria, log_2_ fold change ≥3 or ≤−3, adjusted *P*-value <.1 (Figure 4A). The first part of this filtering strategy identified a list of 71 significant genes, and hierarchical clustering using expression values for these genes, separated TOC and normal esophagus samples (Figure 4B). Moreover, heatmap visualization showed an opposite expression pattern for these genes between TOC and normal samples, and this expression pattern was largely mirrored in sporadic ESCC vs normal RNA-Seq datasets (Figure 4B). These 71 genes were then filtered across the 3 sporadic ESCC vs normal RNA-Seq datasets, revealing a suite of 22 transcriptional changes, 21 downregulated, including *CRISP3*, *IL36A*, and *PSCA*, and 1 upregulated, *KRT17* (further information regarding these genes is described in Table A3). Visualization of these transcriptional changes revealed a similarity between TOC and sporadic ESCC samples (Figure 4C), and 19 of these were subsequently investigated and validated by qPCR (Figure A3). Moreover, some of these genes have been shown to be downregulated at the protein level in ESCC, including PSCA.^33^ Given that *KRT17* was transcriptionally upregulated, it was wondered whether this could be detected at the protein level, and immunofluorescence staining for Keratin 17 was performed. A specific staining pattern localized to papillae structures was observed in normal esophagus tissue, while in TOC esophagus tissue, a diffuse and strong staining pattern was observed throughout all esophageal epithelial cell layers (Figure 4Di), and quantification of mean fluorescence intensity across 9 TOC and 3 normal esophageal samples revealed a trend of upregulated protein expression (Figure 4Dii). Moreover, this matches previously reported Keratin 17 staining in sporadic ESCC,^34^ suggesting this may be an early molecular change in esophageal epithelium.

## Discussion

Data presented here represent the first molecular and transcriptional description of TOC esophagus, with previous studies having focused on iRhom2 biology in the skin and TOC PPK.^5^, ^6^, ^7^ Furthermore, these data may provide novel insights into the early changes that underlie the progression to ESCC.

Numerous transcriptional changes were identifiable in TOC esophageal biopsies that were assumed to be predysplastic relative to normal (wildtype) samples. While some histological aberrations, such as a thickened superficial epithelial layer, were noticeable in TOC esophageal samples (Figure 1A), these data show that many transcriptional changes may occur prior to dysplasia in TOC, which is considered the *bona fide* precursor lesion to ESCC, and several of these changes are also present at the level of sporadic ESCC. Moreover, this indicates that TOC may be a comparable and relevant model to study sporadic ESCC. However, it is not certain whether these changes occur in early-stage predysplastic sporadic cases, as TOC individuals represent a unique ESCC-predisposition cohort, and *RHBDF2* mutations are not known to be found in sporadic ESCCs unlike other familial cancer genes; rather, the genomic loci containing *RHBDF2* is frequently deleted in ESCC.^35^^,^^36^ Considering this, our data still support using TOC to understand sporadic esophageal disease, due to the many similarities observed between TOC and sporadic ESCC (validated across 3 independent datasets), including gene expression changes (Figures 2F and 4B and C), transcription factor predictions (Figure 3D), and enriched pathways (Figures 2Fiii and 3C). Further, it is not currently possible to compare the transcriptomes of TOC with sporadic very early-stage esophageal hyperplasia/dysplasia to identify common early drivers of ESCC, as early-stage ESCC datasets are not available and there are no longitudinal studies where esophageal biopsies have been taken over time before the development of dysplasia and ESCC. This is the unique aspect of studying TOC.

Moreover, replicating this type of study in the sporadic setting would conceivably be difficult to achieve, as to our knowledge, no other patient cohorts are predisposed to ESCC with the same high level of penetrance as TOC. Potential alternative ESCC-predisposition cohorts may include those who live in high-risk regions, such as Zambia^37^ or areas of eastern and central Asia,^1^ or those linked to lifestyle risk factors, such as tobacco smoking and alcohol consumption^38^; although the risk of ESCC in these cohorts is significantly lower than the risk associated with TOC. Moreover, it would be challenging to identify predysplastic sporadic samples that are considered high-risk for ESCC, given that no criteria except for pathology assessment currently provides a readout for disease, and dysplasia is considered the precursor lesion for ESCC.^29^^,^^30^ Therefore, while not directly equivalent, it is suggested that TOC does represent a useful and novel cohort to understand early predysplastic changes in the sporadic setting, and this is supported by data presented throughout this study.

Considering the underlying biology of the TOC esophageal epithelium, changes that might be expected in PPK and skin disease were observed, including enrichment of skin-associated pathways (Figure 3A), upregulation of several keratin genes (Figure 3B), and diffuse and strong staining of Keratin 17 across all esophageal epithelial cell layers (Figure 4D). Given that TOC individuals develop a focal nonepidermolytic PPK and considering that TOC esophageal histology displays an abnormally thickened and keratinized superficial layer, these findings suggest that an abnormal keratinization and keratinocyte differentiation program may be present in TOC esophageal tissue and suggest there may be some similarities between these 2 clinical sites of disease in TOC. Moreover, these changes were apparent across all TOC samples and did not appear to conform to the TOC 1/2 clustering pattern, suggesting these may be constitutive changes linked to iRhom2. Considering this, iRhom2 has previously been shown to associate with the stress keratin, Keratin 16, throughout the keratinocyte stress response,^6^ and given that this and other keratins have been shown to be dysregulated in esophageal epithelium, these may also be linked to iRhom2. Moreover, this suggests that iRhom2 may be a novel and key regulator of multiple keratins in epithelial tissues and links iRhom2 to the sporadic setting, given that Keratin 17 staining in TOC esophageal tissue matches previously reported staining patterns in sporadic ESCC.^34^ It is also plausible that these keratin changes may be mediated by dysregulated EGFR signaling,^39^ downstream of ADAM17 shedding of EGFR ligands, which is facilitated by iRhom2 transport.^9^^,^^10^ However, paradoxically, EGFR1 and TNF-α signaling were predicted to be negatively enriched in TOC esophageal samples (Figures 2Eiii and 3C, respectively), suggesting that these observed changes are not downstream of ADAM17 shedding activity. By contrast, these processes are known to be elevated in TOC skin,^5^ and therefore, these data suggest that TOC and iRhom2 biology in esophageal tissue may be different to the skin and is context specific.

Other considerations include whether iRhom2 is acting to directly or indirectly mediate transcriptional changes in TOC esophageal tissue. It is known that iRhom2 is associated with p63 activity in stressed epidermal keratinocytes^7^; however, our analysis did not predict p63 gene targets to be transcriptionally dysregulated in either TOC 1 or TOC 2 (or ESCC) samples (Figure 3D), suggesting differences between skin and esophageal tissue in TOC. One finding of interest may be the predicted negative enrichment of GRHL2 gene targets (Figure 3Dii and iii) and the perinuclear/cytoplasmic accumulation of GRHL2 protein in papillae structures in TOC esophageal tissue (Figure 3E). Previous work revealed that cytoplasmic localization of a related family member, GRHL3, leads to changes in epidermal differentiation and morphogenesis,^40^ and it may be that cytoplasmic localization of GRHL2 in TOC esophageal tissue drives aberrant differentiation, particularly as this staining pattern was not observed in normal esophageal tissue. Furthermore, a link between p63 and GRHL2 in maintaining a normal epithelial phenotype in keratinocytes has been reported,^41^ and given the link between p63 and iRhom2, we suggest this be explored with future experimentation. Also of note, recent work has found that an N-terminal fragment of iRhom2 can localize to the nucleus and mediate transcriptional changes.^42^ While not explored in esophageal cells or tissue, these findings may link to data presented in this study and could aid understanding of iRhom2 function in esophageal tissue.

Additionally, 22 significant transcriptional changes were identified in TOC and sporadic ESCC samples relative to normal esophageal samples (Figure 4C). These data show that similar transcriptional changes are detectable in samples with limited histological pathology, underscoring TOC as a useful model of sporadic disease. While no validation has been performed using predysplastic sporadic samples, these 22 transcriptional changes may be novel markers of early-stage predysplastic disease, and we propose similar types of studies have potential to identify other markers of esophageal disease.

This study has compared the transcriptomes of TOC and sporadic ESCC and provides a platform for future investigations to reveal early carcinogenic changes in the esophagus. These data also reveal potential effects of iRhom2, a novel familial esophageal cancer gene, in esophageal biology and homeostasis and suggest tissue-specific effects and previously unassigned functions for this pleiotropic protein.
