# Supplementary material for: Investigating iRHOM2-Associated Transcriptional Changes in Tylosis With Esophageal Cancer
Source: Gastro Hep Adv. 2023 Dec 26;3(3):385–95. doi: 10.1016/j.gastha.2023.12.007 (PMC11307647; doi:10.1016/j.gastha.2023.12.007)
Supplement: Supplementary Information [file mmc1.docx]

**SUPPLEMENTARY INFORMATION**

**
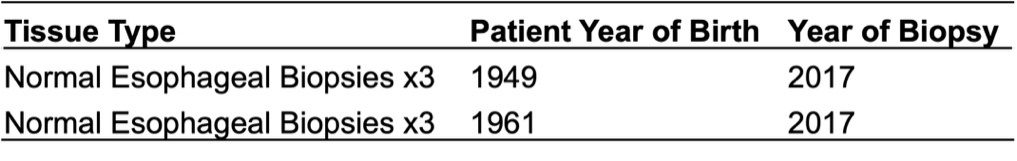
**

**SUPPLEMENTARY TABLE 1. Summary of normal esophageal biopsies used as appropriate controls**

**
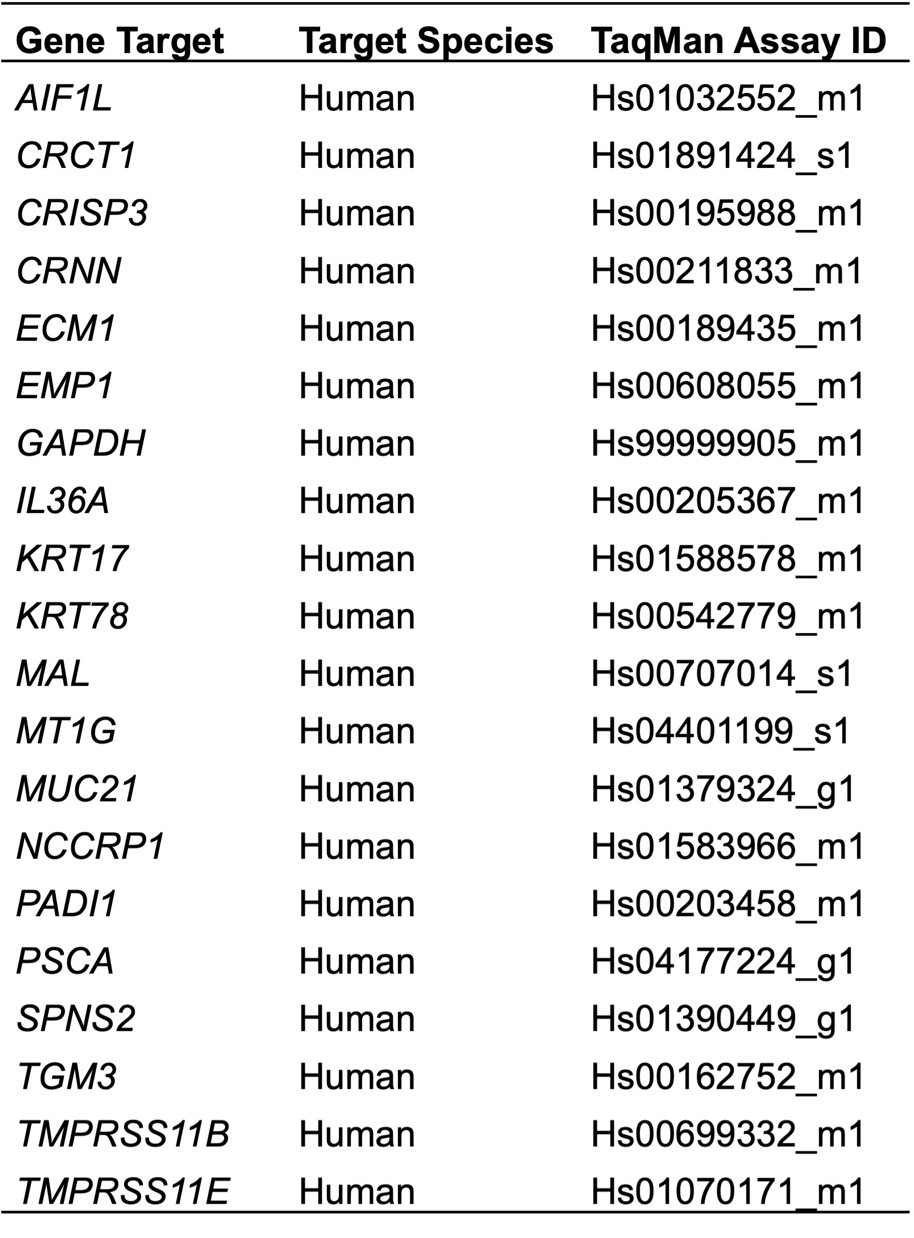
**

**SUPPLEMENTARY TABLE 2. TaqMan assays used throughout study (Supplementary Figure 3)**

**SUPPLEMENTARY TABLE 3. Summary of the 22 genes that are transcriptionally dysregulated in both early-stage TOC and ESCC biopsies (Figure 4C).**

**
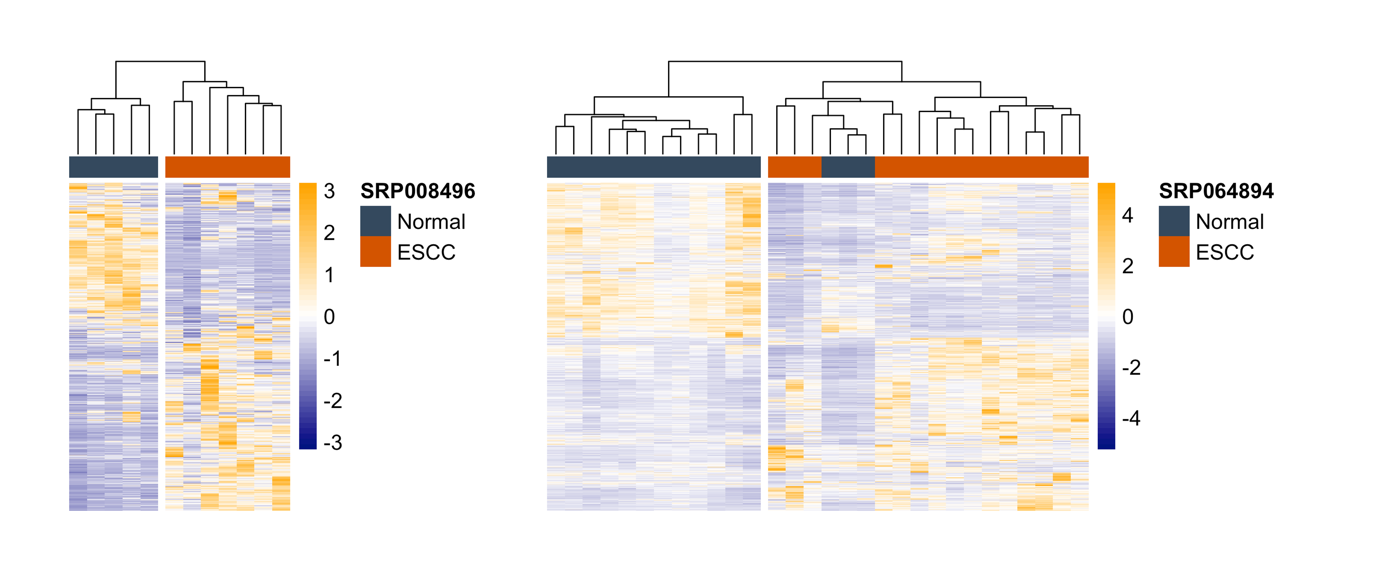
**

**SUPPLEMENTARY FIGURE 1. Heatmaps showing normalised expression counts of 1241 differentially expressed genes for ESCC and control samples in publicly available datasets, SRP008496 and SRP064894.**


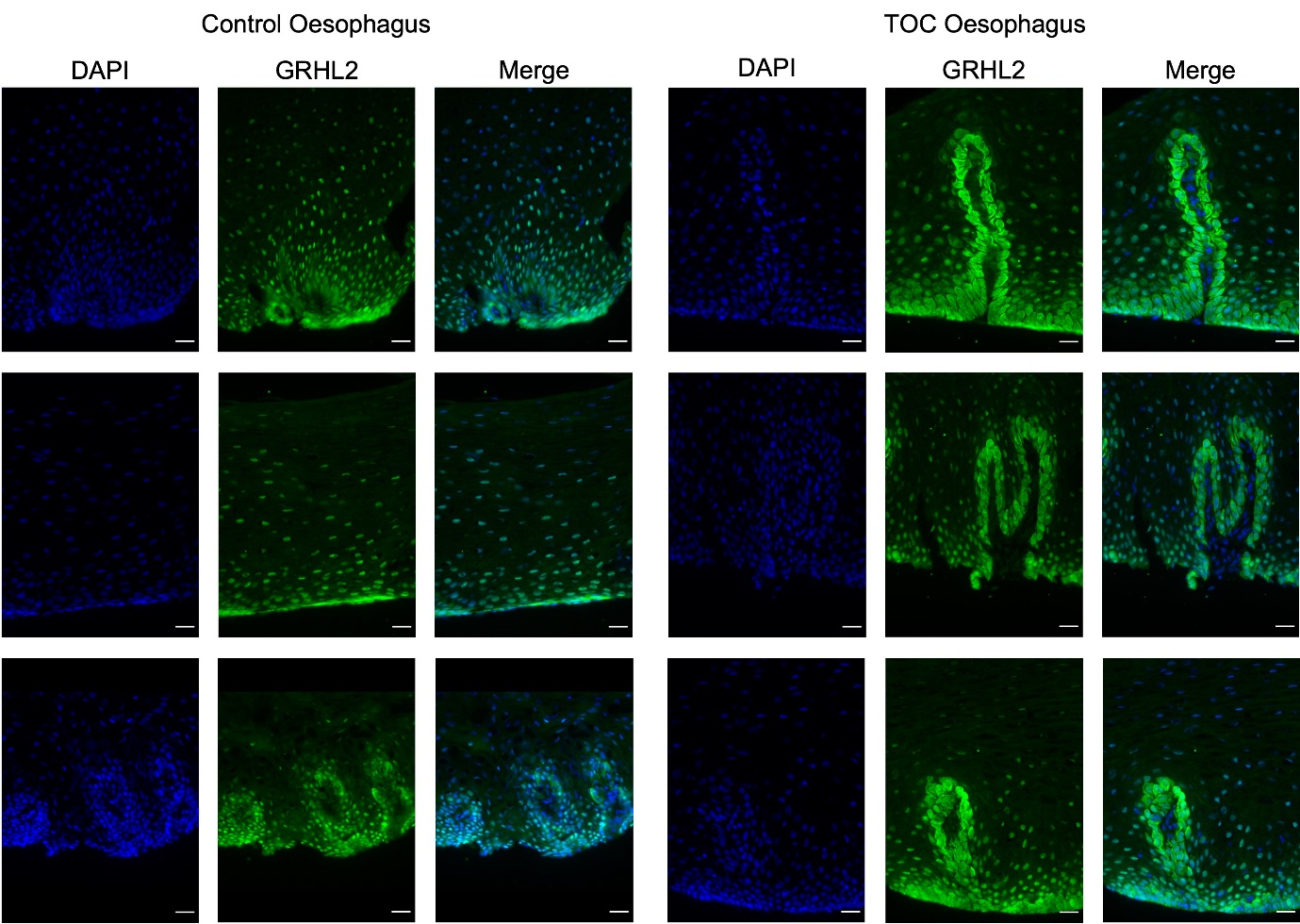


**SUPPLEMENTARY FIGURE 2. GRHL2 localization in control and additional TOC samples.**

GRHL2 is localized in the nucleus in the control samples while in the TOC samples, the localization is nuclear and cytoplasmic (in the furrow regions). Scale bar = 25µm.

**
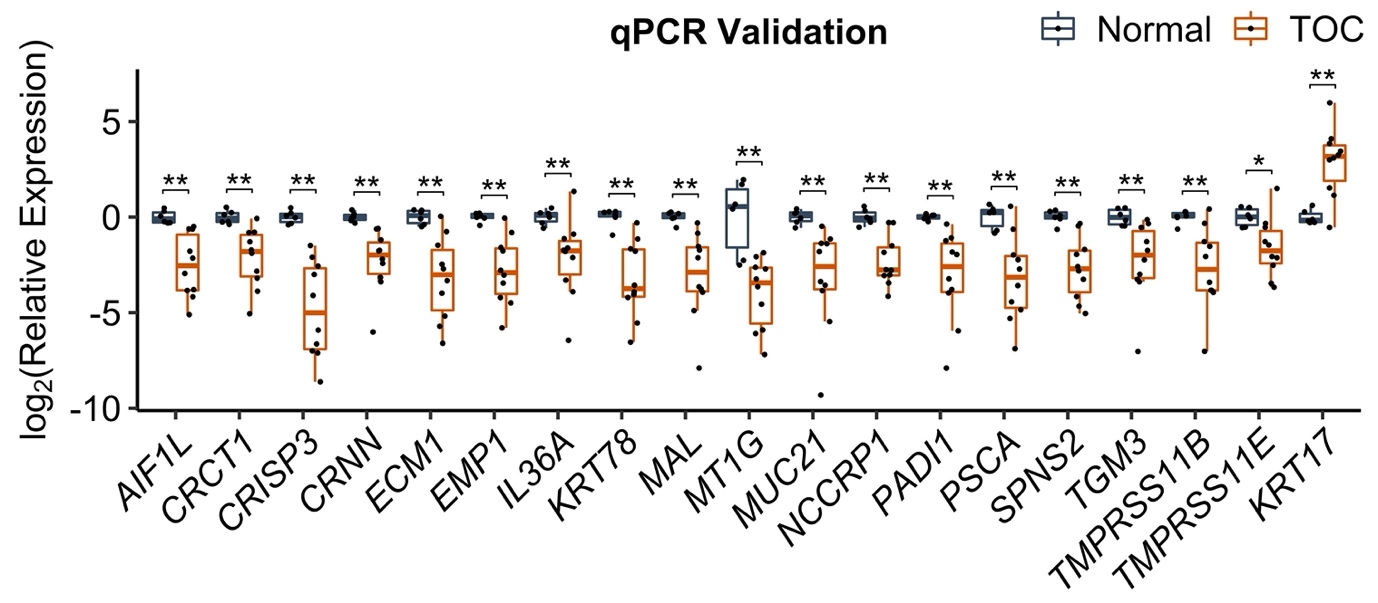
**

**SUPPLEMENTARY FIGURE 3. qPCR validation of 19 gene expression changes in TOC and normal esophageal samples.**

Boxplots showing gene expression (as counts generated from qPCR) of 19/22 candidate genes (identified in Fig. 4C) in TOC and normal esophageal samples. Expression was calculated using the 2^-ΔΔCt^ method, relative to internal GAPDH and to the mean value across normal esophageal samples. Adjusted *p*-values are shown, and statistical significance was calculated using a two-sample t-test with the rstatix package in R (v.0.7.0). ns – Not Significant, *p<0.05, **p<0.01. Plot produced using ggplot2 (v.3.3.5).
